# Supplementary material for: Transplant and Recipient Factors in Prediction of Kidney Transplant Outcomes: A UK-Wide Paired Analysis
Source: J Clin Med. 2022 Apr 15;11(8):2222. doi: 10.3390/jcm11082222 (PMC9024822; doi:10.3390/jcm11082222)
Supplement: Supplementary file 1 [file jcm-11-02222-s001.zip › jcm-1687297-supplementary.pdf]

Supplementary Table S1: pattern of missingness

Variable	**Overall missingness**	**Prolonged DGF, missing data**	**Prolonged DGF, data present**	**Significance (p-value)**
Donor age	0 (0)	-	-	-
Donor sex	0 (0)	-	-	-
Donor BMI	674 (2.8)	67 (9.9)	1043 (4.5)	<0.01
Donor type	0 (0)	-	-	-
Donor retrieval creatinine	2080 (8.8)	146 (7.0)	964 (4.5)	<0.01
Donor history of hypertension	855 (3.6)	58 (6.7)	1052 (4.6)	<0.01
Donor history of diabetes	611 (2.6)	47 (7.7)	1063 (4.6)	<0.01
Donor history of smoking	636 (2.7)	48 (7.5)	1062 (4.6)	<0.01
HLA mismatch group	1 (<0.1)	-	-	-
Cold ischaemic time	244 (1.0)	14 (5.7)	1096 (4.6)	0.53
Recipient age	0 (0)	-	-	-
Recipient sex	12 (<0.1)	-	-	-
Recipient BMI	9300 (39.2)	425 (4.6)	685 (4.8)	0.53
Recipient dialysis modality	100 (0.4)	-	-	-
Recipient diabetes	0 (0)	-	-	-
Recipient wait-time	32 (<0.1)	-	-	-
Re-transplant	0 (0)	-	-	-
Sensitisation	133 (0.5)	-	-	-
Kidney side	0 (0)	-	-	-
HLAi	0 (0)	-	-	-

Variable names (left) are shown alongside percentage missingness for each variable (out of total of 23,700 transplants), and the conditional probability of prolonged DGF given that that variable is observed and not observed. This is shown as the number and percentage of cases of prolonged DGF where that variable was missing, the number and percentage of cases of prolonged DGF where that variable was observed, and the significance of this difference. Differences in prolonged DGF between cases where data was observed vs missing were examined for variables where missing data amounted to greater than 1%.

Supplementary Table S2: demographic details for entire cohort

Variable	**Values**
n	23,700
Donor age, years, mean (sd)	50.05 (14.80)
Donor sex, male (%)	12,737 (53.7)
Donor BMI, units, mean (sd)	26.71 (5.22)
Donor type, DCD (%)	7563 (31.9)
Donor retrieval creatinine, mmol/L, mean (sd)	85.38 (50.43)
HLA mismatch group, n (%)	
1	2993 (12.6)
2	8621 (36.4)
3	10409 (43.9)
4	1676 (7.1)
Cold ischaemic time, hours, mean (sd)	16.29 (5.90)
Recipient age, years, mean (sd)	50.45 (13.27)
Recipient sex, n (%)	14,913 (63.0)
Recipient BMI, units, mean (sd)	26.62 (4.77)
Recipient dialysis modality, n (%)	
HD	14,954 (63.4)
PD	6108 (25.9)
Predialysis	2538 (10.8)
Recipient diabetes, n (%)	2056 (8.7)
Recipient wait-time, years, mean (%)	2.67 (2.41)
Re-transplant, n (%)	3273 (13.8)
Highly sensitised, n (%)	1882 (8.0)
Kidney side, right, n (%)	11,841 (50.0)
HLAi, n (%)	205 (0.9)

Supplementary Table S3: Multivariate Analysis – Odds of prolonged DGF (mismatch number)

Parameter	**Odds ratio (95% CI)**	**p-value for the difference**
Number of HLA mismatches	1.25	0.003
Cold Ischaemia Time (hours	1.07	0.0000006
Waitlist time (years)	1.11	0.01
Recipient age (years)	1.01	0.26
Recipient sex (male)	1.16	0.27
Recipient BMI (units)	1.03	0.04
Recipient dialysis type (PD)	0.54	0.00001
Recipient diabetes	1.59	0.03
Recipient previous renal transplant	2.33	00002
Recipient rejection within 14 days	2.31	0.00008
Recipient highly sensitised	1.34	0.34
Donor organ side (right)	1.06	0.52
HLA incompatible transplant	3.31	0.07

Alternative table 2, where total number of HLA mismatches was considered as opposed to HLA mismatch group.

Supplementary Table S4: paired DGF duration analysis (longer vs shorter)

Parameter	Odds ratio (95% CI)	p-value for the difference
Number of HLA mismatches	1.08	0.01
Cold Ischaemia Time (hours	1.03	<0.0001
Waitlist time (years)	1.06	<0.0001
Recipient age (years)	0.99	0.02
Recipient sex (male)	1.09	0.14
Recipient BMI (units)	1.01	0.09
Recipient dialysis type (PD)	0.54	<0.0001
Recipient diabetes	1.22	0.04
Recipient previous renal transplant	1.47	<0.0001
Recipient rejection within 14 days	1.77	<0.0001
Recipient highly sensitised	1.03	0.82
Donor organ side (right)	1.08	0.06
HLA incompatible transplant	1.98	0.01

Alternative table 2, where the response variable was set as DGF duration (higher or lower) within each donor pair. Primary function was coded as DGF of duration 0 days. This analysis contains data from 6682 transplants where DGF duration was different within each kidney donor pair.

Supplementary Table S5: NHSBT HLA mismatch grade definitions

Level	HLA mismatch summary	HLA mismatch combinations included
1	000	000
2	[0 DR and 0/1 B]	100, 010, 110, 200, 210
3	[0 DR and 2 B] or [1 DR and 0/1 B]	020, 120, 220, 001, 101, 201, 011, 111, 211
4	[1 DR and 2 B] or [2 DR]	021, 121, 221, 002, 102, 202, 012, 112, 212, 022, 122, 222

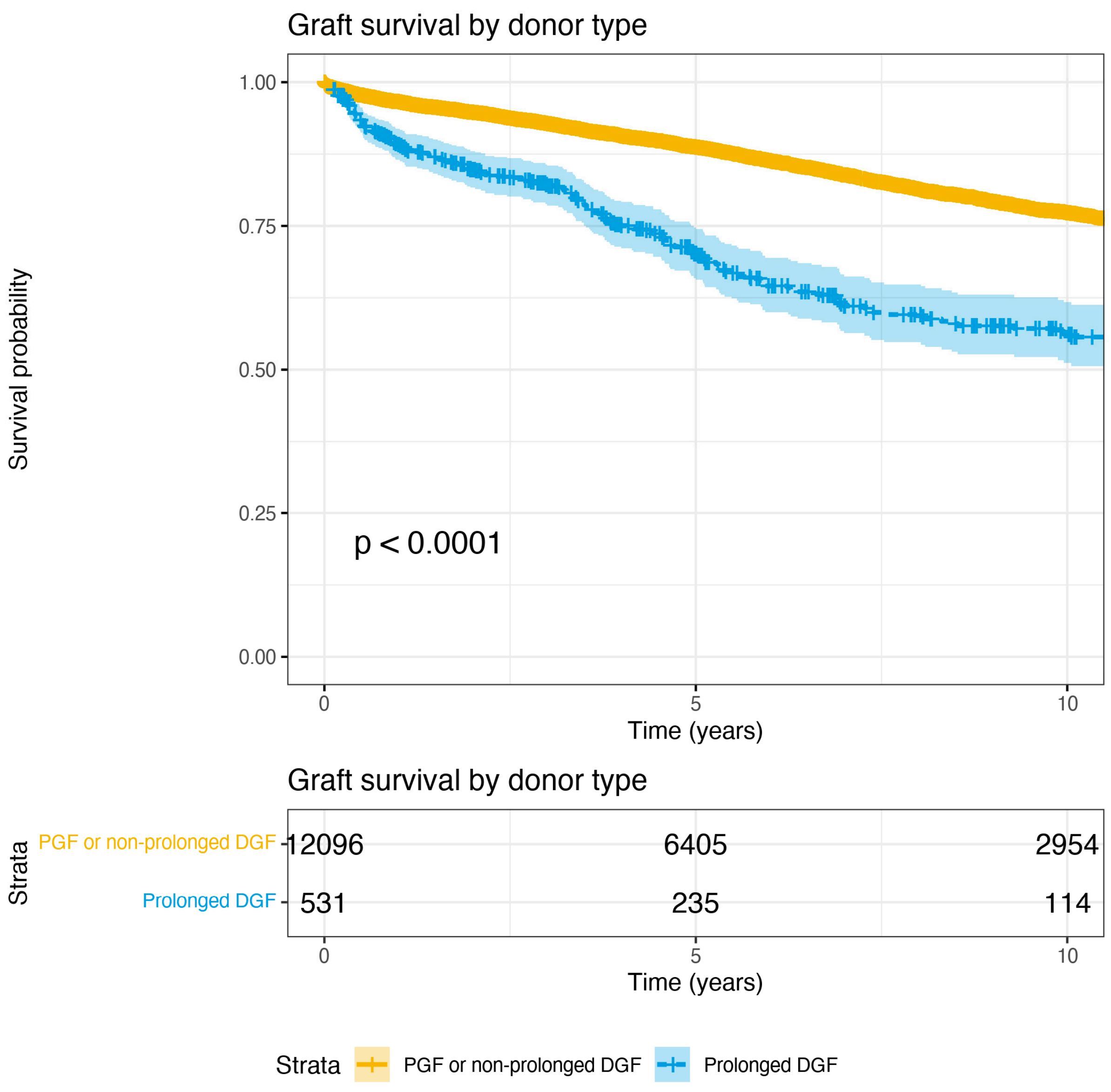

Supplementary Figure S1: Prolonged DGF and graft survival in the DBD cohort

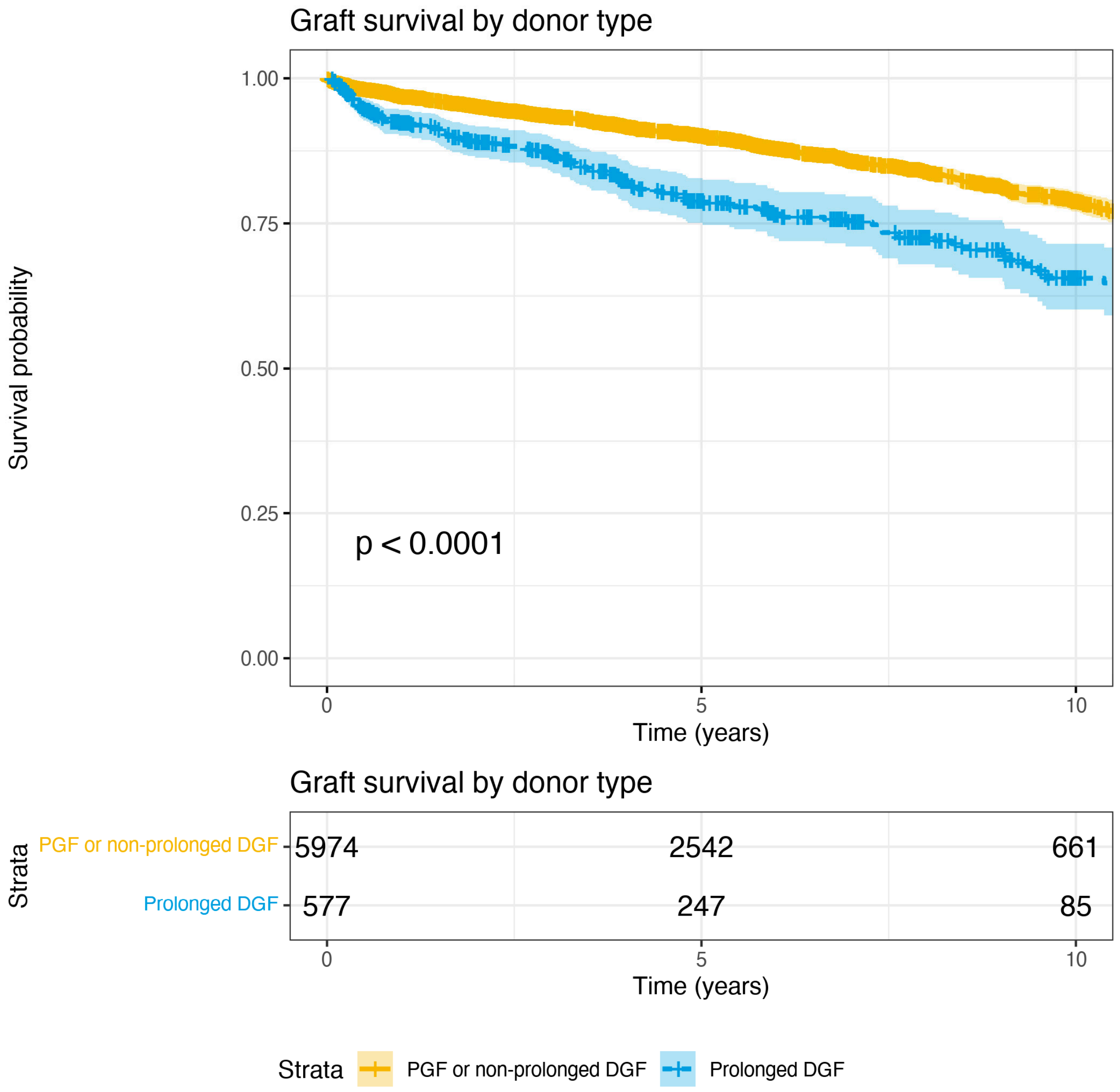

Supplementary Figure S2: Prolonged DGF and graft survival in the DCD cohort
